# Medical Society of the County of Chautauqua

**Published:** 1894-08

**Authors:** C. A. Ellis

**Affiliations:** Secretary


### Society Proceedings.

### MEDICAL SOCIETY OF THE COUNTY OF CHAUTAUQUA.

Reported by C. A. ELLIS, M. D., Secretary.

The Medical Society of the County of Chautauqua, at its annual meeting, held July 10, 1894, elected the following officers: President, Dr. Nelson G. Richmond, Fredonia; vice-president, Dr. Morris N. Bemus, Jamestown; secretary and treasurer, Dr. C. A. Ellis, Sherman.

The scientific part of the program was held at the Hotel Atheneum, Chautauqua, and consisted of an afternoon and evening session.

The guests of the society were Drs. Roswell Park and Lucien Howe, of Buffalo.

The following program was carried out: Topic for discussion, Diphtheria: (1) Its Prevalence, Mortality and Importance of Investigation, Dr. Morris N. Bemus, Jamestown; (2) Observation in Diagnosis, and Some Sanitary Aspects, Dr. J. C. Lewis, Panama; (3) Pathology, Paralysis Following, Dr. O. C. Shaw, Kennedy; (4) Croup and Diphtheria, Unity or Duality, Dr. J. Murphy, Sherman; (5) Intubation—Tracheotomy—Which, When, How Strongly Shall we Urge? Dr. Roswell Park, Buffalo. The referee, who was to read a paper on Treatment, failing to be present, Dr. Park also discussed this part of the subject. (6) Treatment of Purulent Conjunctivitis, Dr. Lucien Howe, Buffalo.

There was a large number in attendance, and a general and free discussion, and great interest manifested in the reading of the papers, which were unusually interesting and instructive.

The society saw fit to postpone indefinitely the consideration of the amendments to the constitution, by-laws, fee bill and code of ethics. These amendments were proposed simply to correct the anomaly which now exists and to harmonize the code and constitution with those of the Medical Society of the State of New York, with which the county society is now in complete affiliation.

---

The best, safest and cheapest sponges are made from absorbent cotton and butter cloth. A bunch of cotton, larger or smaller, as desired, is put on a square of butter cloth, cut the size of a ladies' handkerchief. The four corners are tied over the cotton, and the "sponge" is complete.—*Ex.*

